# Preventing functional loss during immobilization after osteoporotic wrist fractures in elderly patients: a randomized clinical trial

**DOI:** 10.1186/1471-2474-15-287

**Published:** 2014-08-30

**Authors:** Nadja Schott, Heide Korbus

**Affiliations:** Department of Sports and Exercise Science, University of Stuttgart, Allmandring 28, 70569 Stuttgart, Germany

**Keywords:** Distal radius fracture, Mental practice, Mirror therapy, Immobilization, Women ≥ 60 years, Clinical trial

## Abstract

**Background:**

Distal radius fractures are among the most common fractures and account for approximately one-sixth of all fractures diagnosed. Therapy results after distal radius fracture, especially of elderly patients, are often suboptimal. The inevitable immobilization for several weeks leads to reduction in range of motion, deterioration of muscle strength, malfunction of fine motor skills as well as changes of motor and sensory representations in the brain. Currently, there are no strategies to counteract these immobilization problems. The overall aim of the study is to investigate the therapeutic potential of motor-cognitive approaches (mental practice or mirror therapy) on hand function after wrist fracture.

**Methods/Design:**

This study is a controlled, randomized, longitudinal intervention study with three intervention groups. One experimental group imagines movements of the fractured upper extremity without executing them (mental practice). The second experimental group receives a mirror therapy program consisting of the performance of functional movement synergies using the unaffected forearm, wrist, and hand. The control group completes a relaxation training regime. Additionally, all patients receive usual care by the general practitioner. We include women aged 60 years and older having a distal radius fracture and sufficient cognitive function. All groups are visited at home for therapy sessions 5 times per week for the first 3 weeks and 3 times per week for weeks 4 to 6. Measurements are taken at therapy onset, and after 3, 6 and 12 weeks. The primary outcome measure will assess upper extremity functioning (Patient-Rated Wrist Evaluation [PRWE]), while secondary outcome measures cover subjective wrist function (Disabilities of the Arm and Shoulder; [DASH], objective impairment (range of motion, grip force) and quality of life (EuroQol-5D, [EQ5D]).

**Discussion:**

Results from this trial will contribute to the evidence on motor-cognitive approaches in the early therapy of distal radius fractures.

**Trial registration:**

The trial is registered at ClinicalTrials.gov with registration number NCT01394809 and was granted permission by the Medical Ethical Review Committee of the University of Tübingen in June 2011.

**Electronic supplementary material:**

The online version of this article (doi:10.1186/1471-2474-15-287) contains supplementary material, which is available to authorized users.

## Background

Distal radius fractures are among the most common osteoporotic fractures[1] and account for an estimated 17% of fractures treated in US emergency departments[2] with a female–male ratio of about 3:1[3]. Corresponding to the demographic development, osteoporotic fractures of the wrist, humerus spine or hip can be expected to increase further in the coming years and with it the burden on healthcare resources[4, 5]. Regardless of the fact whether these fractures are treated surgically or by casting, patients are at least immobilized for two to six weeks or more. Physical and occupational therapy as a key element in rehabilitation starts after the removal of the fixation device. However, during the period of fixation, patients often keep their injured hand in rigid postures, in which the volar plates and adjacent ligaments of the digital joints are shortened[6]. Different methods of treatment, but especially the long immobilization periods lead to overall complication rates ranging from 6 to 80% and have been associated with poor functional outcomes[7]. These complications not only include complex and regional pain syndrome, stiffness, nerve injury, tendon and ligament injuries, but a massive reduction in range of motion (ROM), muscular atrophy, and loss of movement representation[8]. As a result, the final hand function is often suboptimal[9]. Previous studies have indicated that 20% of patients with distal radius fracture had persistent symptoms, and 10% continued to have functional impairments after the typical recovery period[10]. In a study by consortium partners we demonstrated that the risk of losing independence after a wrist fracture is almost as high as after a hip fracture[11]. This partly relates to upper extremity dysfunction with activities of daily living such as eating, getting dressed and washed.

The goal for rehabilitation after wrist fractures is to achieve complete and rapid recovery of ROM, strength, and function of the wrist and hand. Improvement of the functional outcome after wrist fracture can probably not only be found in changing the operative technique[12]. Hence, for improvement of functional outcome, one has to focus on the postoperative rehabilitation period[13, 14]. A patient would need a treatment procedure that is more active without actually stressing the bone and that may prevent from the negative side effects as well as from the central reorganization that takes place as a result of immobilization. This leads to a temporary forgetting of the function of the affected limb[15], and results in the inefficiency of the central control of movements. Immobilization has shown to result rather rapidly in changes of motor and sensory representations in the brain of peripheral organs such as finger, arm, or leg[16, 17]. For example, Langer et al.[16] showed a decrease in cortical thickness in the left primary motor and somatosensory area as well as a decrease in the grey matter in the left corticospinal tract after at least 14 days of limb immobilization.

Several studies have shown that sensory input does not exclusively result from actually performed movements. Imagined movements without actually moving the limbs (explicit motor imagery) as well as observational learning (mirror therapy) also generate sensory input[18–21]. Mirror therapy (MT), in which a mirror is placed in the patient's midsagittal plane, so that he/she can see his/her unaffected arm/hand as if it was the affected one, has mainly been studied for two different purposes: pain relief[22], and motor recovery post-stroke[23–25]. Furthermore, MT has been shown to increase ipsilateral primary motor excitability in healthy controls[26], which may account for the improvement in motor function. Mental practice (MP) represents a class of training or therapy regimes in which an internal representation of a movement is repeatedly simulated in mind from a first-person perspective, without actual physical movement, and is effective in motor recovery in neurological and orthopedic rehabilitation[27–29]. Proposed mechanisms for improved motor recovery with MT and MP include reconciliation of motor output and sensory feedback MT,[30] and graded activation of cortical motor networks MP,[31]. According to Jeannerod[32, 33] MP and the preparation of movements share common mechanisms and are functionally equivalent[34]. Furthermore, the activation of pre-motor “mirror neurons”, which have intimate connections with visual processing areas, is thought to prime the primary motor cortex and to be important in imitating motor action[35–37].

In orthopedic rehabilitation MP as well as MT has only received minor attention as a promising psychological complement to conventional physical therapy approaches. Three studies have examined the effects of MT in patients after hand surgery other than amputation[18, 38, 39]. While Rosén and Lundborg[38] reported three different cases (e.g. tendon repair) when MT was applied in combination with traditional hand training (no further details regarding the number of training sessions or duration were given), Altschuler and Hu[18] as well as Rostami et al.[39] examined hand function with different orthopedic conditions in one (insufficient information on intervention), respectively 12 patients (15 sessions à 30 minutes). Both studies conducted MT in combination with physical therapy. All three studies reported improvements in objective as well as subjective measures of hand function. None of these studies had the goal to overcome the effects of immobilization. There are mixed results on the effects of MP during disuse or immobilization[40–45]. Some researchers have found support for mental imagery to maintain muscle strength and flexibility[42, 44] Schott & Limberger: Effects of mental practice on gait after total hip endoprosthesis, in prep], while others have not[40, 41, 43, 45]. Methodological inconsistencies could account for many of the contradictory findings within the literature. Studies vary in the participants’ age and health status, the type of imagery, the content of imagery, as well as intervention length. Explicit imagery has been shown to create the greatest physiological benefits but only few studies specifically elaborate on their imagery script. Protocols for imagery intervention also varied among researchers from as little as 10 days to 7 weeks in duration with varying numbers of sessions per day. To our knowledge, only two studies examined the efficacy of explicit motor imagery and MT so far[42, 46]. Ietswaart and colleagues[46] found no enhanced improvement as a result of MP with motor imagery in upper hand function of stroke patients. Frenkel[42] examined the efficacy of a MP combined with MT after knee endoprothesis surgery, but only found significant results for the criterion flexion. No other studies exist with regard to patients with orthopedic injuries, comparing the efficacy of MT or MP.

The purpose of this randomized study is to determine whether explicit motor imagery or MT during the immobilization period after distal radius fracture results in a greater recovery of central aspects of hand function. The objective of this study is to establish the effectiveness of daily training of movement imagery with the affected arm (MP) or the healthy arm (MT). It will also be examined whether enhanced functional recovery of the hand due to MP or MT is also associated with increases in the amount of activities of daily living. It will be investigated whether the benefit of motor-cognitive approaches for distal radius fracture patients is related to their individual differences in motor imagery ability.

## Methods/Design

Post-fracture strategies that tackle the problems mentioned above might reduce the functional loss after wrist fractures and improve quality of life considerably. Currently, no proactive strategies are published to counteract the immobilization problems. Our participants are being recruited from different hospitals and randomly allocated to one of three groups. There are two experimental intervention groups (MP and MT) and one control group. We use two different strategies originally developed and tested for the rehabilitation of stroke patients: Participants either receive a MP or a MT program. This novel approach combines neuropsychological approaches with sport science and conventional physiotherapy.

The study is conducted as a randomized controlled trial. The purpose of the study is to examine the effects of MP and MT on upper extremity function after osteoporotic wrist fractures.

The trial is conducted in agreement with the principles of the Declaration of Helsinki, and with the guidelines of Good Clinical Practice (GCP). The study protocol was approved by the local and independent Ethics Committee Tübingen (Institutional Review Board).

### Procedure

**Figure 1:**
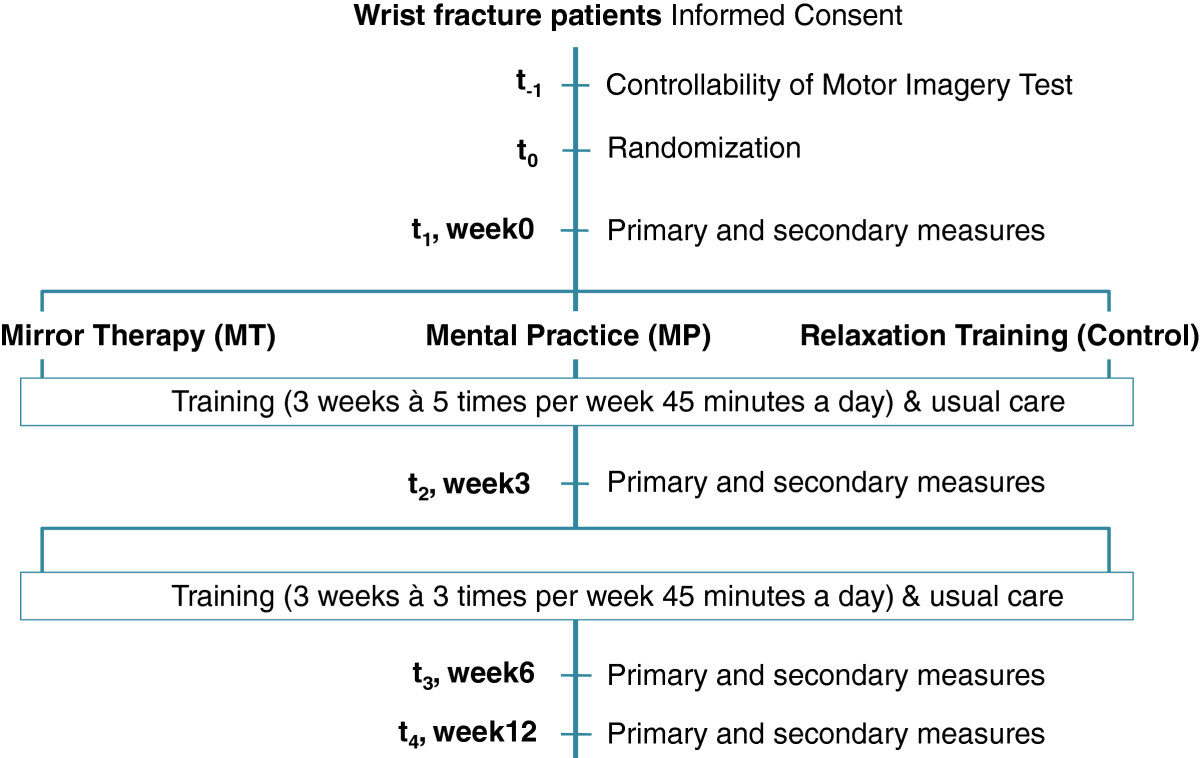
**Flow diagram of study design.**

All patients give written informed consent prior to the study. Before randomization, the patients complete the Controllability of Motor Imagery Test[47] to control for the ability to perform and control a mental image of a movement, which is essential for successful imagery training. After baseline assessment, randomization is performed by an independent department (Department of Biometrics at Ulm University).Assessment of primary and secondary outcomes takes place upon entry to the study (T1) by a blinded assessor and is repeated after 3 weeks (T2), 6 weeks (T3), and 12 weeks (T4, follow-up) after the beginning of the training program (see Figure 1).

### Participants

We include females aged 60 years and older with a distal radius fracture. We exclude persons with the following characteristics:

- Unstable medical conditions which preclude surgical intervention (ASA 5)
- Patients with an open fracture
- Associated soft tissue or skeletal injury to the same limb
- Cognitive impairment 6CIT[48],

### Intervention

Participants in the experimental groups receive either a MP or a MT program. Patients in the control group are instructed to practice relaxation techniques. The intervention period lasts six weeks and takes place in the patient’s private home. Additionally, all patients receive therapy as usual. All MP and MT training sessions were provided by the same physical therapist; however, a second therapist conducted the primary and secondary evaluations. In week one to three the patient participates in five sessions per week on five consecutive days (Monday-Friday), and in week’s four to six the patient participates three times per week with the therapist and two times without. As of week 4, when the guided therapy sessions are reduced to 3 times per week, all patients receive written instructions containing descriptions and photos of the movement tasks. They are asked to document their training sessions (duration and level of difficulty) in daily training diaries. Additionally, the patients are supplied with an MP3-player with a text for progressive muscle relaxation. All therapy sessions are 45 minutes per treatment session. Each session (MP as well as MT) starts with a standardized progressive muscle relaxation focusing on wrist (healthy side) and other body parts (based on[49]), and is applied for five to ten minutes to aid the participant’s ability to concentrate on the internal body sensations of the following imagination protocol.

The content of the two motor-cognitive interventions (MP and MT) is based on a simplified version of Gentile’s task taxonomy[50]: four sections are defined by the combination of stationary/mobile performer with open/closed environment. The basic movement tasks in this study are dorsal extension, palmar flexion, radial and ulnar abduction as well as supination and pronation using Gentile’s Taxonomy of Tasks to structure the training content and progression ([51]; details provided in Table 1). Object manipulation and/or intertrial variability mark higher levels of difficulty. Patients are asked to rate the level of difficulty for mentally simulating the movement, respectively for perceiving the reflected image in the mirror as their affected side (1 very easy to 10 very hard). As soon as they rate a task with 4 (“reasonably easy”) or lower, they try a task of a higher difficulty level. For retaining same conditions, the patients should not execute the movement with the affected side during MP and MT.

**Table 1:** **Movement tasks based on Gentile’s taxonomy of tasks**[51]

		Body stability	
		No object manipulation	Object manipulation
		⇨ dorsal extension	⇨ lifting up weight (dorsal extension)
		⇨ palmar flexion	⇨ lifting up weight (palmar flexion)
		⇨ ulnar-/radial abduction	⇨ wiping a table with a cloth
		⇨ supination/pronation	⇨ handling of a bike lock/kitchen timer
	**No intertrial variability**	⇨ opening and closing hand (like grasping a cup)	⇨ wringing out a towel
		⇨ thumb-finger-oppositions	⇨ compressing a softball
		⇨ finger-tapping sequence	⇨ grasping a pen with little finger (e.g.) and thumb
			⇨ playing piano (simple 5-finger-scale)
**Stationary environment**			
		⇨ Dorsal extension against different resistances	⇨ grasping, lifting, turning different cups (size, weight)
		⇨ Palmar flexion against different resistances	⇨ catching different balls on a marple run (reaction)
		⇨ Ulnar-/radial abduction against different resistances	⇨ compressing softballs of different sizes/hardness
	**Intertrial variability**	⇨ Supination/pronation against different resistances	⇨ pulling a cloth across the table (ulnar/radial) against different resistances
		⇨ Swimming in turbulent water	⇨ bowling/rolling a ball towards a partner
			⇨ playing ping-pong
			⇨ juggling with one hand

**Figure 2:**
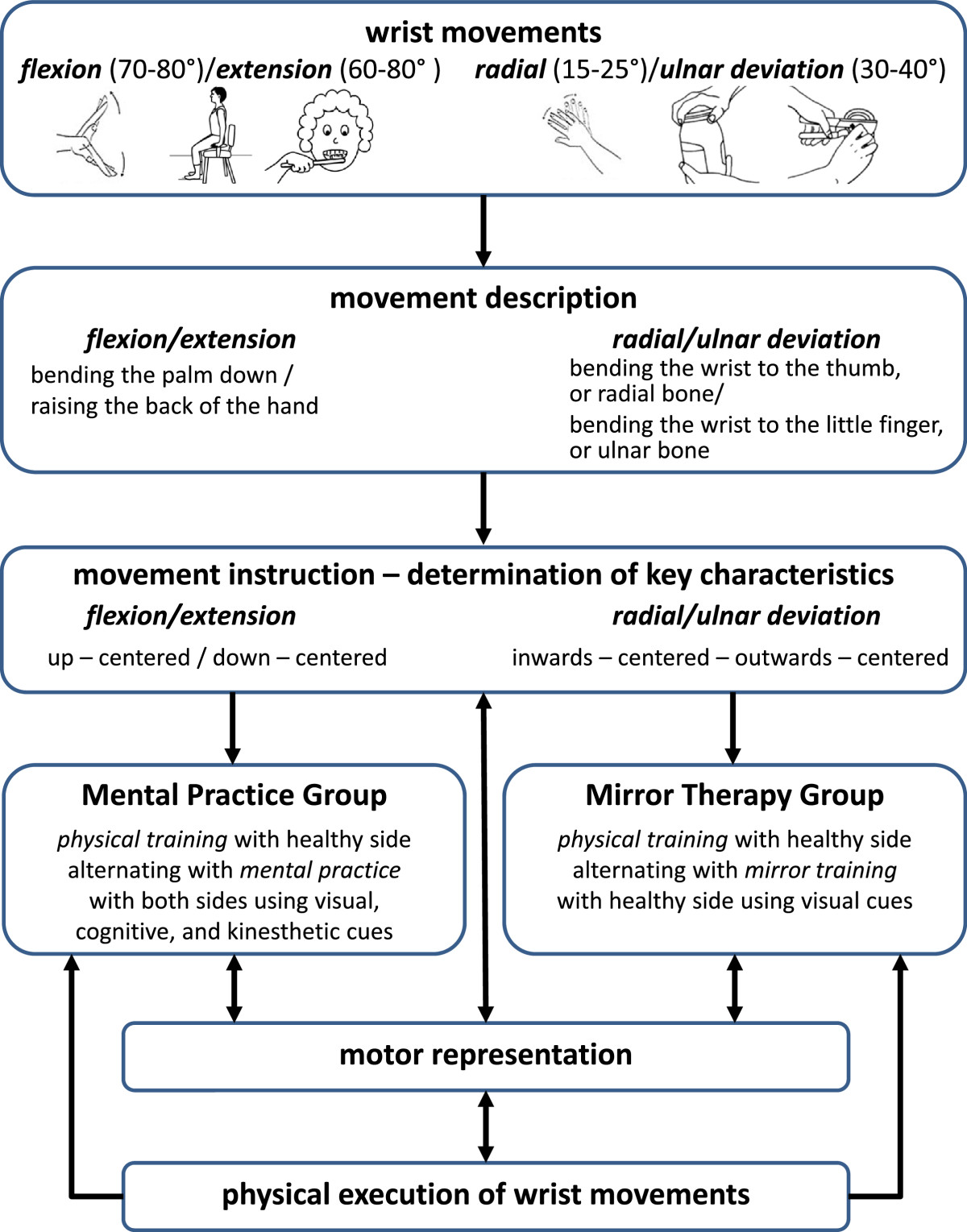
**MT and MP procedure after distal radius fracture (modified from**[43, 52, 53]**).**

The training protocol for MT and MP after distal radius fracture follows the standardized method of the Mental Gait Training[43, 52]. It consists of several sequential steps: (1) description and (2) instruction of wrist movements; (3) the physical training with the unaffected side in alteration with either MP (with both wrists) or MT (only with the unaffected side); (4) building and strengthening of the motor representation of wrist movements, and (5) the physical execution of wrist movements (providing that the actual sensation does not disturb the mental image) (see Figure 2).

In a first step the therapist discusses with the patient wrist movements the patient executes on a typical day. Both develop a therapy plan together in order to define which movements should be addressed during therapy. In the next step the patient is confronted with the physiological model of wrist movements, the movement descriptions. A movement description is the objective, biomechanical representation of the wrist movement using here the unaffected hand of the patient as a model. This method is used to provide the relevant functional aspects of the reference movement. Additionally, in order to achieve greater learning and retention results, the patients are prompted to verbalize the movements from an individual inside view addressing as many sensory modalities from memory as possible. The next step consists of the elaboration of the key characteristics of the different wrist movements. Finally, the key characteristics are marked symbolically, i.e. they are renamed as individual short formulae (e.g. up – centered or pull - release for dorsal flexion). The aim is the compression of the information (chunking). The image should be approximated thus to the dynamism and the temporal duration of the real movement[54].

After finalizing the movement descriptions the patients in the MP group execute movements with their unaffected side to provide additional feedback such as posture, muscle tension, or joint positions. The physical training distinguishes itself by a sort of proprioceptive training in which under modification of perception, movement and environment the movement representation should be further stabilized and differentiated. The key characteristics for flexion were *up-centered*, and for the ulnar and radial deviation *inwards-centered-outwards-centered*. Following the physical training with open and closed eyes to enhance kinesthetic perception, the patients in the MP group imagined normal wrist movement with the unaffected side with different imagery modalities (kinesthetic, visual; for examples see Table 2) with the following sequence (modified from[43]):

**Table 2:** **Examples of mental practice script delivered to MP group**

Visual:	How does your hand look turning a door key? Imagine seeing your hand, the key, and the door. See your wrist turning and the forearm pronating.
**Kinesthetic:**	Imagine touching the cold metal of the key with your fingers. Now feel your muscles in your forearm and hand activate as you grasp and turn the key. What can you feel? Is there a tension in your muscles?

1. Active execution with the unaffected side (1 x with open eyes)
2. Visual imagery (1 x unaffected side, 1 x affected side; with closed eyes)
3. Active execution with the unaffected side (1 x with closed eyes)
4. Kinesthetic imagery (1 x unaffected side, 1 x affected side; with closed eyes).

After establishing the internal concept of wrist movements, the systematic training phase with physical training alternating with MP follows this protocol:

1. Active execution with the unaffected side (2 x with open eyes)
2. Active execution with the unaffected side (2 x with closed eyes)
3. Kinesthetic imagery with the unaffected side (1 x with closed eyes)
4. Kinesthetic imagery with the affected side (5–10 x with closed eyes)

Due to the findings of Yae et al.[55], that mental imagery is most effective with a kinesthetic focus, our training process is aiming at that modality.

Patients in the MT group immediately start with the observation of the movements with the unaffected side in the mirror. During MT the affected hand remains hidden from view behind the mirror, which is placed in the midsagittal plane of the participant. The patient is observing the reflected image of the unaffected hand, which appears like the affected side performing the movement. Patients perform each wrist movement with the unaffected side 8 to 10 times with visual focus on the mirrored picture of the healthy side.

The overall aim of these protocols is to preserve motor representation of hand function to enable patients to execute wrist movements with high accuracy and appropriate strength after splint removal.

The control group receives relaxation training to achieve the same total amount of time the therapist spends with the patients of the experimental groups.

### Outcome measures

The assessments focus on changes in three key domains: subjective wrist function (PRWE-G; DASH), objective impairment (Range of motion, grip strength) and quality of life/social engagement (DASH, EuroQol).

#### Primary outcome

Is the subjective rating of pain and impairment in activities of daily life (*Patient-rated Wrist Evaluation; PRWE-G*, German version,[56]), a validated tool for assessing functional outcome in patients with distal radius fracture[57]. The questionnaire is completed by the investigator via patient interview and consists of two domains, pain and function. There are five items in the pain domain (e.g. “Rate your pain when lifting a heavy object.”) and ten items in the function domain (e.g. “Rate your difficulty you experience when fastening buttons on your shirt.”). The response to each item is scored on a scale of 0 to 10. Scores of each individual item were provided with qualitative descriptors defined as: none (0), minimal (1–2), mild (3–4), moderate (5–6), severe (7–8) or very severe (9–10). The pain score is the sum of five items, with the worst possible score of 50, and the disability (function) score is the sum of ten items divided by 2.

#### Secondary outcomes

The *Disabilities of the Arm, Shoulder and Hand (DASH*;[58]) score is a 11-item, self-administered questionnaire designed to measure physical functions, symptoms, and social function, work, sleep, and confidence items in patients with any or several musculoskeletal disorders of the upper limb. The DASH outcome measure is scored in two components: the Disability/Symptom and the optional high performance Sport/Music module. The DASH Disability/Symptom score is a summation of the responses to 11 questions on a scale of 1 (without difficulty or no symptom) to 5 (unable to engage in activity or very severe symptom). This value is then transformed to a score out of 100 by subtracting one and multiplying by 25 with values between 0 (no disability) and 100 (severe disability). The questions examine the degree of difficulty in performing a variety of physical activities because of arm, shoulder, or hand problems (6 items). It also investigates the severity of pain, tingling (2 items), as well as the effect of the upper limb problem on social activities, work, and sleep (3 items).

*Range of motion (ROM)* of the radiocarpal joint is measured on both sides with a handheld goniometer. Four parameters are evaluated: dorsal extension, palmar flexion, radial and ulnar abduction of the wrist joint[59].

*Grip strength* is measured with a handheld dynamometer (Myon, Prophysics), and carried out three trials on each side. If necessary, the instrument is adjusted to the size of the patients’ hands. The patient is sitting in a chair, her upper arm by her side of the body and the forearm stretched to an angle of 90°, with the elbow supported. The patient is encouraged to squeeze the dynamometer as hard as possible. A mean value is determined according to the standardized Mathiowetz procedure[60]. The grip strength is presented as the percentage of the value of the injured side of the value of the uninjured side. In order to account for hand dominance in grip strength, if the non-dominant hand was injured, the percentage will be multiplied by 1.10; if the dominant hand was injured, the percentage will be multiplied by 0.90[61].

Social engagement is evaluated with the social functioning subscale of the DASH-Questionnaire[58] and health related quality of life with the EQ-5D[62]. The EQ-5D is a short questionnaire with five dimensions (mobility, self-care, usual activities, pain or discomfort, and anxiety or depression), each of which can be rated at one of three levels (1 no problem, 2 some problems, 3 extreme/severe problems). EQ-5D utility scores range between a full health score of 1 (where the respondent has no problems on any dimension) and the lowest score of -0.59 (when the respondent reports that they are at the bottom level of each dimension).

Additionally, falls associated self-efficacy is evaluated (ABC-Scale, German version,[63]). Demographic information is analyzed at the beginning of the intervention period.

### Sample size/power calculations

The sample size calculation was approximated with a 3×3-factorial ANOVA-approach based on the primary outcome. Previous randomised controlled trials of therapy interventions have shown effect sizes ≥ .80 in similar populations for the PRWE as a result of hand therapy, however therapy starting weeks after the accident. To prove an intervention effect with about medium effect size of Cohen's f = 0.25 with an error probability α = 0.05, a power β = 0.8, a correlation between repeated measures of .05, and a nonsphericity correction at 1, n ≈ 12 people in each study arm are required for analysis. The analysis was carried out with the G*Power software (G*Power V 3.1.6 Franz Faul, Universität Kiel, Kiel, Germany). To account for potential attrition, we will inflate the sample size by 10%, resulting in a total sample size of 39 with 13 participants allocated to each group.

### Statistical analysis

Data will be analyzed using (M)ANOVA with repeated measures to ascertain the effects of therapy on outcome variables and to follow improvements individually in time. An Intention-to-treat principle will be applied. Missing data will be replaced by an imputation method for missing measurements.

## Discussion

This trial is due to deliver results by the end of 2014. Up to now, participants have shown a very high compliance, less than 10% resigned (1 in MP group, 1 in CG). Recruitment is more difficult than expected, partly caused by a very short hospital stay combined with the dependency of prompt communication of new adequate patients, partly due to an exceptionally mild winter (less falls), and partly due to communication deficits. Therefore, the intervention period turns out to be several months longer than initially planned.

## Conclusions

Study outcomes should be carefully analyzed, possibly leading to further adjustments of the intervention protocol or to a second trial that is ruling out the assumed reasons for inconclusive results like a high rate of intercurrent diseases. A positive study outcome should be tested in a large multi-center trial.

## Authors’ original submitted files for images

Below are the links to the authors’ original submitted files for images. Authors’ original file for figure 1 Authors’ original file for figure 2
