# Supplementary figures and images for: Preventing functional loss during immobilization after osteoporotic wrist fractures in elderly patients: a randomized clinical trial
Source: BMC Musculoskelet Disord. 2014 Aug 30;15:287. doi: 10.1186/1471-2474-15-287 (PMC4158045; doi:10.1186/1471-2474-15-287)

# Wrist fracture patients Informed Consent

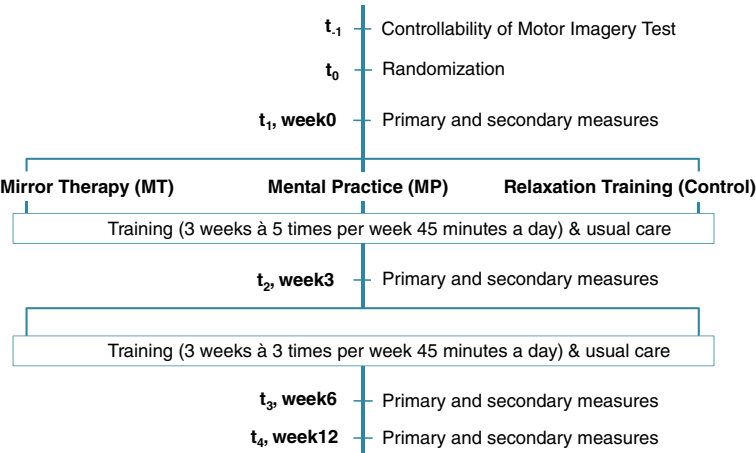

Supplement: Supplementary file 1 — Authors’ original file for figure 1 [file 12891_2014_2232_MOESM1_ESM.pdf]
