# Supplementary material for: Preventing functional loss during immobilization after osteoporotic wrist fractures in elderly patients: a randomized clinical trial
Source: BMC Musculoskelet Disord. 2014 Aug 30;15:287. doi: 10.1186/1471-2474-15-287 (PMC4158045; doi:10.1186/1471-2474-15-287)
Supplement: Supplementary file 2 — Authors’ original file for figure 2 [file 12891_2014_2232_MOESM2_ESM.pdf]

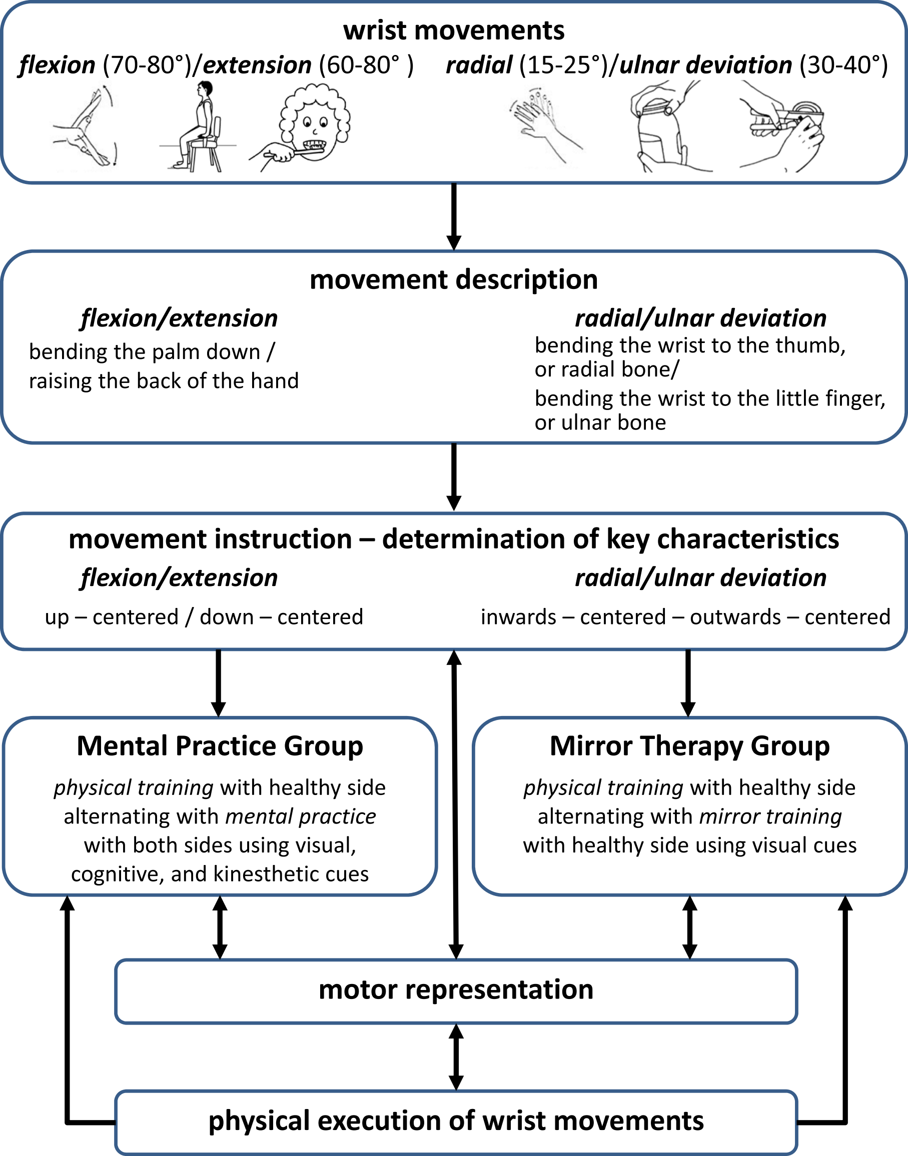
wrist movements
flexion (70-80°)/extension (60-80°)
radial (15-25°)/ulnar deviation (30-40°)
movement description
flexion/extension
bending the palm down /
raising the back of the hand
radial/ulnar deviation
bending the wrist to the thumb, or radial bone/
bending the wrist to the little finger, or ulnar bone
movement instruction – determination of key characteristics
flexion/extension
up – centered / down – centered
radial/ulnar deviation
inwards – centered – outwards – centered
Mental Practice Group
physical training with healthy side alternating with mental practice with both sides using visual, cognitive, and kinesthetic cues
Mirror Therapy Group
physical training with healthy side alternating with mirror training with healthy side using visual cues
motor representation
physical execution of wrist movements
